# Cerebral Venous Sinus Thrombosis Complicating Middle Ear Infections: A Rare Complication in Post-Antibiotic Era

**DOI:** 10.7759/cureus.18964

**Published:** 2021-10-22

**Authors:** Sarvesh C Mishra, Isha Tyagi, Aviral Gupta, Srishti Sharma, Lavanya Tyagi

**Affiliations:** 1 Radiodiagnosis, Sanjay Gandhi Post Graduate Institute of Medical Sciences, Lucknow, IND; 2 Neuro-otology, Sanjay Gandhi Post Graduate Institute of Medical Sciences, Lucknow, IND; 3 Pathology, Sanjay Gandhi Post Graduate Institute of Medical Sciences, Lucknow, IND; 4 Obstetrics and Gynecology, Javitri Hospital and Test Tube Baby Centre, Lucknow, IND

**Keywords:** cerebral venous sinus thrombosis (cvst), otomastoiditis, post-antibiotic era, contrast enhanced magnetic resonance imaging (cemri), low molecular weight heparin (lmwh)

## Abstract

In the post-antibiotic era, intracranial and extracranial complications of middle ear infections have become rare. Similarly, cerebral venous sinus thrombosis (CVST), a frequent complication of middle ear infections, has become rare now. Here, we present a case of a 27-year-old male who presented with a short history of severe headache and associated episodes of intractable vomiting. There was also a prior history of right ear discharge one year back which responded to medical management. The patient did not improve clinically even after prompt symptomatic management. Contrast-enhanced magnetic resonance imaging (CEMRI) of the head and contrast-enhanced magnetic resonance venogram (CEMRV) were done, which showed right-sided otomastoiditis complicated with CVST and meningitis. Although the clinical signs of meningeal irritation and mastoid tenderness were not present on clinical examination. The patient was started on anticoagulant therapy and antibiotics for two weeks following which there was marked clinical improvement.

## Introduction

Cerebral venous sinus thrombosis (CVST) used to be a very dreadful complication of middle ear infection in the early 20th century with decreased incidence and associated mortality in the post-antibiotic era. However, recently due to the indiscriminate usage of antibiotics and the resultant emergence of antibiotic-resistant strains, has led to an increase in the cases of acute and chronic mastoiditis complicated by CVST. Inherited hypercoagulable states are a major risk factor in young adults. Here, we present a case of a young adult male, who presented with CVST. The patient presented with a history of severe headaches with vomiting. There was a history of right ear discharge one year back, which resolved to medical management and the patient was asymptomatic from last one year. During the investigation, he was found to have mastoiditis for which he was not having any symptoms complicated by CVST. So this case highlights the importance of having a high index of suspicion of CVST in a patient with otomastoiditis coming with a headache. Prompt diagnosis and treatment can result in a significant reduction of mortality as well as morbidity. In this case report, we have described the diagnostic approach for such a patient presenting with a headache along with a relevant review of the literature.

## Case presentation

A 27-year-old male nurse presented to the emergency department (ED) with complaints of severe holocranial headache for three days with associated intractable vomiting. Three days back the patient woke up in the afternoon with a severe headache following which he became unconscious. The patient regained consciousness after about 5 minutes when he developed the aforementioned episodes of vomiting. There was no history of trauma, fever, weakness over one side of the body, or visual disturbances. There is no history of addiction, pulmonary Koch’s infection, or hypothyroidism. The patient was not on any antihypertensive medication.

However, there was a history of episodes of right ear discharge one year back for which he took some medication and was symptom-free for the past one year. Clinical examination showed raised blood pressure of 140/90mm Hg with a pulse rate of 86/minute. The patient was afebrile with no pallor, icterus, lymphadenopathy, or peripheral edema. The respiratory system was within normal limits. No focal neurological deficits or cranial nerve palsies. There was the presence of right-sided mastoid tenderness with absent signs of inflammation in the overlying skin. There was no active ear discharge on external ear examination. 

The patient was admitted for further evaluation and symptomatic treatment for headache was started in the form of antiosmotic agents and antiemetics for vomiting. Even after prompt symptomatic treatment using analgesics, antiemetics, antiacids, and IV fluids, there was no significant clinical improvement following which contrast-enhanced magnetic resonance imaging (CEMRI) along with contrast-enhanced magnetic resonance venogram (CEMRV) were done which revealed right otomastoiditis complicated by acute thrombosis of the right transverse and sigmoid sinuses along with meningitis. The patient was started on low molecular weight heparin (LMWH) and parenteral antibiotics.

He was also investigated for inherited and acquired hypercoagulable states. There was marked clinical improvement and the patient was discharged with the suggestion of follow-up in the neurology outpatient department (OPD). He was asymptomatic at one-month follow-up and was advised mastoidectomy as per surgical opinion from ENT surgeon.

Investigations

Complete blood count (CBC) was normal with Hb 13gm/dl. Total leucocyte count (9400 per microliter), differential leukocyte count (neutrophils 62%, lymphocytes 31%, monocytes 3%, eosinophils 3%, and basophils 1%), and platelets (3.1 lakhs per microliter) were within normal limits. Erythrocyte sedimentation rate (ESR) was raised (49mm/h, normal range < 15mm/h) and prothrombin time (PT) was normal (INR: 1.14). Serum creatinine and C-reactive protein were normal, antinuclear antibody and antiparietal cell antibodies were negative, and antiphospholipid antibody profile was within normal limits. Lupus anticoagulant and activated protein C resistance assay were negative, vitamin B12 level was normal, homocysteine and folate were within normal limits, paroxysmal nocturnal hemoglobinuria (PNH) screen was negative, antithrombin-III activity level and protein C functional assay were marginally abnormal with antithrombin activity level measuring 129% (normal range: 80-120%) and protein C levels measuring 131% (normal range: 70-130%), normal protein S-functional assay with protein S activity level measuring 85% (normal range: 70-140%), and mildly increased plasma fibrinogen level measuring 439mg/dl (normal range: 200-400mg/dl).

**Figure 1:**
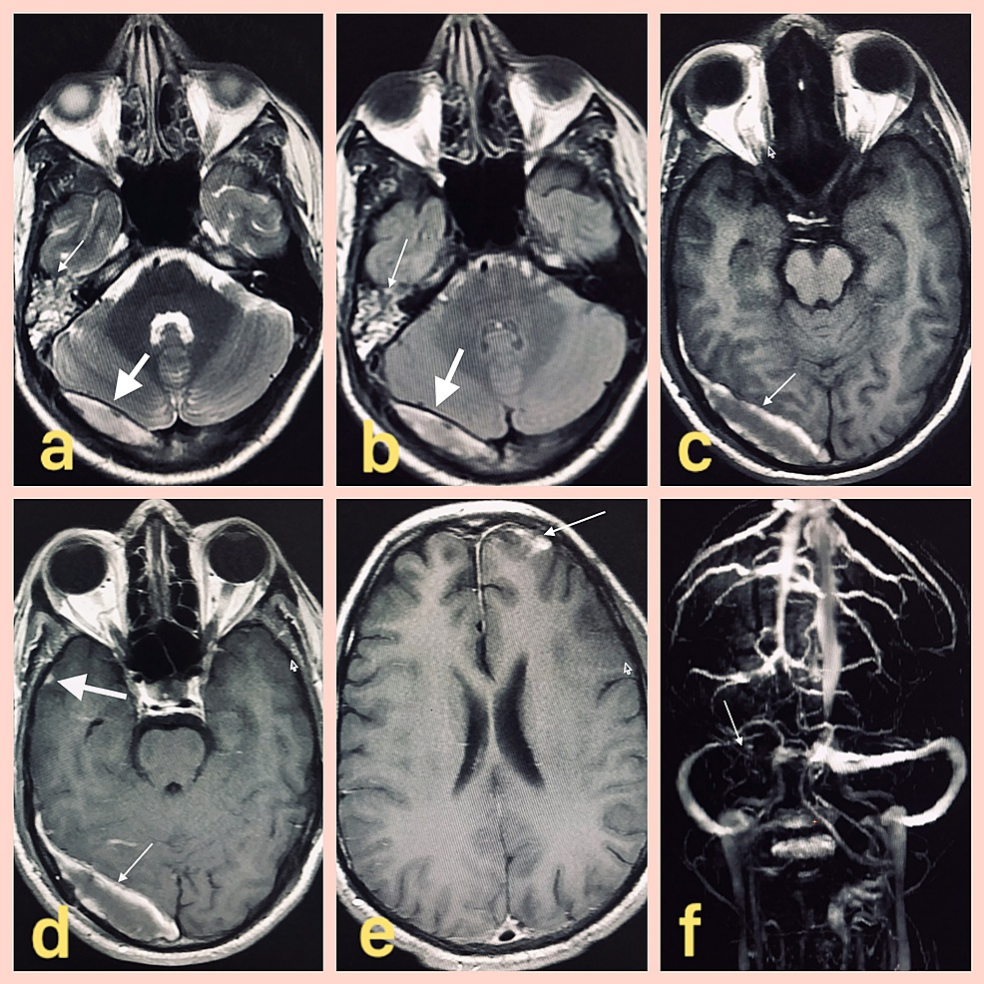
Axial T2WI, T2-FLAIR, T1 non-contrast, T1WI post-contrast, 3D TOF-MRV images of the brain. (a) Axial T2WI of the brain showing fluid intensity in the right mastoid air cells (denoted by thin white arrow) and T2 hyperintense distended right transverse sinus (denoted by thick white arrow). (b) Axial T2 FLAIR of the brain showing similar signal characteristics involving the right mastoid air cells (denoted by thin white arrow) and right transverse sinus (denoted by thick white arrow). (c) Axial T1 non-contrast image showing distended right transverse sinus (denoted by white arrow). (d) Axial T1WI post-contrast image showing a non-enhancing filling defect in the right transverse sinus (denoted by thin white arrow) and a focus of abnormal parenchymal enhancement in the right temporal lobe (denoted by thick white arrow). (e) Axial T1WI post-contrast images showing a nodular focus of leptomeningeal enhancement in the left frontal lobe (denoted by thin white arrow). (f) 3D TOF-MRV image showing the non-visualization of the right transverse sinus and portion of the right sigmoid sinus (denoted by white arrow). T2WI: T2-weighted image; T1WI: T1-weighted image; T2-FLAIR: T2-weighted-fluid-attenuated inversion recovery; TOF-MRV: time of flight-magnetic resonance venography

CEMRI with CEMRV showed fluid intensity in right mastoid air cells with distended hyperintense right transverse and sigmoid sinuses showing loss of normal flow void on T2 and T2-weighted-fluid-attenuated inversion recovery (T2-FLAIR) (Figures 1a, 1b). On axial T1 non-contrast scan, there was evidence of distended right transverse sinus, and on axial T1 post-contrast scan, there was non-enhancing filling in the distended venous sinus suggestive of acute thrombosis (Figures 1c, 1d). There was an associated focus of nodular leptomeningeal enhancement in the right temporal lobe and left frontal lobe suggestive of meningitis (Figures 1d, 1e). There was a non-visualization of the right transverse on the 3D time of flight angiography-magnetic resonance venography(TOF-MRV) image (Figure 1f). 

The patient was given parenteral antibiotics for meningitis. Injection low molecular weight heparin (LMWH) was given twice daily, subcutaneously followed by a course of oral anticoagulant dabigatran etexilate. Oral antiepileptics were given to prevent seizures. The patient responded to the treatment well and was discharged in good general condition. The patient was on follow-up in neurology OPD and show marked improvement in the neurological symptoms related to CVST and is now dated to undergo radical mastoidectomy as per consultation with the ENT department.

## Discussion

The intracranial and extracranial complications of middle ear infections have decreased significantly in the post-antibiotic era. CVST, one of the complications frequenting middle ear and mastoid infections has also become rare. The mortality associated with CVST reached 100% in the early 20th century [1] and has now decreased significantly to less than 10% due to the widespread availability of highly potent antibiotics [2]. CVST can have associated morbidity due to septic cardiomyopathy, acute respiratory distress syndrome (ARDS), anacusis, and seizures [3]. However, due to indiscriminate use of antibiotics and antibiotic resistance, the cases are still seen complicating the acute and chronic middle ear and mastoid infections [4]. In young adults, hypercoagulable states both inherited (protein C and protein S deficiency, factor V Leiden mutation, etc.) and acquired (trauma, tumor, inflammation, pregnancy, etc.) are a major risk factor [5,6]. Infections involving the middle ear structures such as acute otitis and mastoiditis uncommonly cause thrombosis of the sigmoid and transverse sinuses due to structural contiguity [7,8]. There is a direct spread of the inflammatory process from the middle ear infections including acute otomastoiditis via the small venues draining into the sigmoid sinus [9]. The thrombosis of the cerebral venous sinuses causes intracranial venous hypertension [8,10]. The occlusion of the veins also leads to cerebral edema and venous infarction. CSVT can have varied clinical manifestations, wherein 30% can present acutely within two days of blockage, half of the patients present in a sub-acute fashion within two days to 30 days, and 20% may present anytime from 30 days to six months [10]. Around nine out of 10 adult patients diagnosed with CVST had ipsilateral headache [8,10]. Other presentations include edema and tenderness over the mastoid process called as “Griesinger sign,” nausea, vomiting, altered mental status, seizures, focal motor deficit, double vision, and earache [8-10]. Intracranial hypertension leading to papilledema and resultant visual deficits has been reported in 13.2% of the patients [7,9,10]. Ophthalmoplegia can occur secondary to third, fourth, and sixth cranial nerve palsy with associated eye tenderness [8-10]. Uncontrolled intracranial hypertension can cause major complications such as permanent blindness, status epilepticus, coma, and death from cerebral herniation [8]. In cases of middle ear infection with suspected intracranial complications, imaging is imperative for confirming diagnosis and planning management. CT head with temporal bone can show erosive changes in the bones, empyema, cerebral abscess, and also the thrombus in the cerebral venous sinuses which manifests radiologically as a “delta sign” [3]. MRI head with contrast MRV is the most sensitive modality for confirmation of CVST [8,11]. The thrombosed venous sinuses show low or absent flow and clot formation, which appears as increased signal intensity in T1 and T2 images and the presence of inflammation in the brain and meninges [9,11]. MRI can be normal in three out of 10 patients [10]. However, CT venography and MRI venography have 95% sensitivity [8,10,11]. Of late, the treatment of CVST has become more conservative [12]. Once the diagnosis of CVST is established, anticoagulation initiation with heparin is crucial [8,10]. Heparin causes clearing of the thrombus from occluded cerebral veins/sinuses, reverses the thrombotic process as well as prevents further thrombus propagation, and prevents pulmonary embolism [8,11]. Unfractionated (UFH) and low molecular weight heparin (LMWH) are used with LMWH preferred over UFH due to practical advantages [10]. Routinely, the duration of anticoagulation is three months, but three to six months for impermanent risk factors such as trauma, pregnancy, infection, etc. A longer duration of anticoagulation up to 12 months is required for pro-thrombotic states, e.g., active malignancy [10,11]. In patients not responding to medical management and showing progressive worsening, endovascular interventions (thrombolysis and venoplasty) can be done, and in cases with increased intracranial pressure causing pressure effects, decompression craniotomy can be done [10]. With the advancements in neuroimaging, prompt diagnosis and early treatment have markedly decreased the mortality associated with CVST with good long-term neurological outcomes [13]. A high index of suspicion and early diagnosis using modalities like CEMRV and CT can be lifesaving. Thorough workup should be done in young adult patients to rule out associated coagulation pathologies. Undiagnosed CVST can have a fulminant clinical course with increased intracranial hemorrhage and its complications like edema and mass effect.

## Conclusions

A high index of suspicion should be kept for evaluating cerebral venous sinus thrombosis in the setting of otomastoiditis with headache and vomiting. CVST complicating otomastoiditis is usually seen due to the emergence of antibiotic-resistant strains or due to coagulation disorders in young patients. They should be kept on long-term anticoagulation once the diagnosis is confirmed. Parenteral antibiotics play a pivotal role in arresting the infective/inflammatory process. MRI with CEMRV is very useful in confirming the diagnosis. Workup should be done to rule out conditions associated with increased blood coagulability and deranged coagulation profile, especially in young patients.
